# Supplementary material for: Understanding antimicrobial use in subsistence farmers in Chikwawa District Malawi, implications for public awareness campaigns
Source: PLOS Glob Public Health. 2022 Jun 8;2(6):e0000314. doi: 10.1371/journal.pgph.0000314 (PMC10021458; doi:10.1371/journal.pgph.0000314)
Supplement: S1 Text — (DOCX) [file pgph.0000314.s001.docx]

Standards for Reporting Qualitative Research (SRQR)

| **No** | **Element** | **Reported in the paper** |
| --- | --- | --- |
| **Title and abstract** | | |
| S1 | Title | P1 |
| S2 | Abstract | P2 |
| **Introduction** | | |
| S3 | Problem formulation | P3, para 1 |
| S4 | Purpose or research question | P8, para 2 |
| **Methods** | | |
| S5 | Qualitative approach and research paradigm | P8,para 1 and 2 |
| S6 | Researcher characteristics and reflexivity | P9 para 2 |
| S7 | Context | P5 para 3 – P7 para 1 |
| S8 | Sampling strategy | P9 para 2 |
| S9 | Ethical issues pertaining to human subjects | P11 para 2 |
| S10 | Data collection methods | P8 para 2 – p10 para 2 |
| S11 | Data collection instruments and technologies | Supplementary file with structured data collection tool |
| S12 | Units of study | P9 para 1 (structured medicine interviews)  P9 para 2 (ethnographic fieldwork)  P10 para 2 (interviews) |
| S13 | Data processing | P10 para 2 and 3 |
| S14 | Data analysis | P10 para 3 – p11 p1 |
| S15 | Techniques to enhance trustworthiness | P10 para 3 |
| **Results and findings** | | |
| S16 | Synthesis and interpretation | P11-23 |
| S17 | Links to empirical data | P11-23 |
| **Discussion** | | |
| S18 | Integration with prior work, implications, transferability, and contribution(s) to the field | P23-P25 |
| S19 | Limitations | P26 |
| **Other** | | |
| S20 | Conflicts of interest | P27 |
| S21 | Funding | P26 |
