# Supplementary material for: Understanding antimicrobial use in subsistence farmers in Chikwawa District Malawi, implications for public awareness campaigns
Source: PLOS Glob Public Health. 2022 Jun 8;2(6):e0000314. doi: 10.1371/journal.pgph.0000314 (PMC10021458; doi:10.1371/journal.pgph.0000314)
Supplement: S2 Text — (DOCX) [file pgph.0000314.s002.docx]

| **VARIABLE** | **FREQUENCY/PERCENTAGE** |
| --- | --- |
| 1. **Interviewer name:** |  |
| 1. **Date of interview:** |  |
| 1. **Household Number:** |  |
| 1. **Village** *(To be typed in)*   **Mudzi** |  |
| 1. **Are you able to locate the household head or a primary caregiver? Yes/No**   **Kodi mutu wabanja panyumba pano ndi ndani?** | If no, end here |
| 1. **Has the adult consented to the survey? Yes/No**   **Kodi munthu wamkulu wapeleka chilolezo kuti mukhoza kupanga kafukufuku? Inde/ayi** | If no, end here |
| 1. **GPS coordinates of household** |  |
| 1. **Duration they have lived in the area**   **Nthawi imene akhala ku dera**  Months________ (if less than a year)  Years _________(if more than a year) |  |
| **RESPONDENT CHARACTERISTICS** |  |
| 1. **Role of respondent in the household**   **Udindo wa munthu yemwe akuyakha mafunso panyumba**  Head of household  Mutu wa banja  Primary caregiver  Wopeleka chisamaliro  Child  mwana  Relative  M’mbale  Friend  Mzake  Other (Specify)  Zina( fotokozani) |  |
| 1. **Sex of respondent**   Male  Female |  |
| 1. **Age of respondent (In absolute numbers)**   **Zaka za munthu woyakha mafunso** |  |
| 1. **Highest level of education of respondent**   **Maphunziro munafika nawo patali bwanji**  1 Never went to school  2 Primary education  3 Malawi primary leaving certificate  4. Secondary school  5. Malawi school certificate of education  6.. Diploma  7. Vocational training  8. University degree  9. Other (Specify)  10. Refused to answer |  |
| 1. **Primary occupation of respondent**   **Ntchito yodalilika imene mukugwira**  1 -Unemployed  2- Farmer  3 – Bicycle/motorcycle taxis driver  4 -Market vendor  5- Tailor  6 -Builder/Mason  7-Carpenter  10. Refused to answer  11. Government employee  12. NGO employee  13. Sugar worker  14. Other (specify) |  |
| 1. **Ethnicity of respondent**   **Mtundu wa munthu woyankha mafunso**  Mang’anja  Sena  Tambuka  Lomwe  Yao  Chewa  Other (Specify) |  |
| **ILLNESSES AND MEDICINES** | |
| 1. **Illnesses frequently experienced in the household (Tick all that apply) Matenda omwe mumadwala kawirikawili panyumba pano**   Malaria  Cough/Cold/flu  Diarrhoea  Abdominal pain  Ulcers  Headache  High blood pressure  Sickle cells  Skin rashes/Eczema  Typhoid  Other (Specify) |  |
| 1. **Medicines used to treat the frequently experienced illnesses in the household**   **Mankhwala amene mumagwilitsa ntchito kuchiza matenda amene mumadwala kawirikawiri pa nyumba pano**   - - Medicines for Malaria   - Medicines for Cough/Cold/flu   - Medicines for Diarrhoea   - Medicines for abdominal pain   - Medicines for Ulcers   - Medicines for Headache   - Medicines for Skin rashes/Eczema   - Medicines for Typhoid   - Other(Specify) |  |
| 1. **Medicines stored in the household (Tick all that apply)**   **None Mankwala amene mumasunga pa nyumba pano**  Medicines for Diabetes (Capture the name of the medicine)  Medicines for Diarrhoea (Capture the name of the medicine)  Medicines for Hypertension (Capture the name of the medicine)  Medicines for Pain (Specify the pain) (Capture the name of the medicine)  Headache  Stomach pain  Joint pain  General body pain  Muscle pain  Medicines for abdominal problems. (Capture the name of the medicine)  Medicines for Cough/’Flu’/Pneumonia. (Capture the name of the medicine)  Medicines for HIV/ISS/AIDS. (Capture the name of the medicine)  Medicines for Asthma. (Capture the name of the medicine)  Medicines for Skin rashes/Eczema. (Capture the name of the medicine)  Medicines for Malaria. (Capture the name of the medicine)  Medicines for ulcers. (Capture the name of the medicine)  Medicines for typhoid. (Capture the name of the medicine)  Other( specify medicine and capture the name of the medicine) | (***Provision for taking the photo of the medicine stored***) |
| 1. **Sources of medicines commonly used in the household (Tick all that apply) Komwe mumapeza mankhwala amene mukugwilitsa ntchito pa nyumba pano**   Drug shop  Pharmacy  Public Health Facility  Private clinic  Private hospital  Research clinic/NGO  Traditional healer  Family/friends  Don’t know  Other (specify) |  |
| **ANTIBIOTICS USED IN THE HOUSEHOLD Pile sorting exercise** | |
| **We would now like to continue asking you about medicines. To do this, we would like to show you some medicines that we have brought with us. (Present the antibiotic library to participant). Doctors often call these kinds of medicine ‘antibiotics’. We would like to do some exercises with you, where we will ask you to sort these medicines into different piles. The exercises are not to test your knowledge, but to find out whether you have seen any of these before and which you have used. The reason that we are doing the exercises is that it helps to actually show you these medicines to help you remember which ones you have seen. As you will see, the exercises do not take very long. If you are OK to continue, we will begin.**  ***Tsopano ndipitiliza kukufunsani za mankhwala. Ndikuwonesani mulu wa mankhwala amene ndabweresa. (Wonesani antibiotic library kwa wotenga nawo gawo mu kafukufuku) A za umoyo amawatchula mankhwala awa kuti ma antibiotics. Ndikupemphani kuti muyike mankhwala amenewa mu milu. Izi sizikuchitidwa pofuna kuyesa nzeru zanu, koma kudziwa ngati munawawonapo kapena kugwirisa ntchito. Chimene tikukuwoneserani mankhwalawa ndi kufuna kukuthandizani kukumbukira amene munawawonapo. Muwona kuti sititenga nthawi yayitali. Ngati mukuvomereza tikhoza kupitiliza***   1. **Antibiotics recognised (Tick all that apply)**   **Mankhwala amene mukuwadziwa**  For each pile sorting exercise, encourage the participant to narrate to you the stories behind the relevant piles (e.g. the story of not being able to access a particular medicine).  *Pa kusanthula mulu uliwonse alimbikiseni wotenga nawo mbali mukafukufuku kuti azikufotokozerani nkhani zokhuzana ndi milu imene akuwunjika [mwachitsanzo nkhani yovutika kupeza mankhwala amene amawafuna]*   1. **Sorting Medicines activity one:** Which of these medicines have you seen or heard of before? Please pick out the ones that you have seen or heard of before and put them into one pile.   **Zochitika zoyamba posanthula mankhwala:** *Ndi mankhwala ati amene munawawonapo kapena kumva za iwo? Chonde sankhani amene munawawonapo kapena kumvapo ndikuwayika pa mulu umodzi.*   \| 1. Amoxicillin (Mapilisi) \| \| --- \| \| 2. Amoxicillin (Amadzi) \| \| 3. Ampicillin (Mapilisi) \| \| 4. Azithromycin (Mapilisi) \| \| 5. Benzathene Penicillin (Obaya) \| \| 6. Benzylpenicillin (Obaya) \| \| 7. Ceftriaxone (Obaya) \| \| 8. Cefuroxime (Mapilisi) \| \| 9. Cefalexin (Mapilisi) \| \| 10. Cefixime (Mapilisi) \| \| 11. Chloramphenicol (Mapilisi) \| \| 12. Chloramphenicol (Obaya) \| \| 13. Ciprofloxacin (Mapilisi) \| \| 14. Clarithromycin (Mapilisi) \| \| 15. Clavulinic acid/ amoxicillin (Mapilisi) \| \| 16. Clindamycin (Mapilisi) \| \| 17. Clindamycin (Obaya) \| \| 18. Cloxacillin (Mapilisi) \| \| 19. Cloxacillin (Obaya) \| \| 20. Cotrimoxazole (Mapilisi) \| \| 21. Cotrimoxazole (Amadzi) \| \| 22. Doxycycline (Mapilisi) \| \| 23. Erythromycin (Mapilisi) \| \| 24. Erythromycin (Amadzi) \| \| 25. Flucloxacillin (Mapilisi) \| \| 26. Flucloxacillin/amoxicillin (Mapilisi) \| \| 27. Gentamycin (Obaya) \| \| 28. Levofloxacillin (Mapilisi) \| \| 29. Metronidazole (Mapilisi) \| \| 30. Metronidazole (Amadzi) \| \| 31. Metronidazole (Obaya) \| \| 32. Norfloxacin/ metronidazole (Mapilisi) \| \| 33. Ofloxacin/ Ornidazole (Mapilisi) \| \| 34. Phenoxymethylpenicillin (Mapilisi) \| \| 35. Tetracycline (Mapilisi) \| \| 36. Chloramphenicol (Mmaso/Kukhutu) \| \| 37. Ciprofloxacin (Mmaso) \| \| 38. Gentamicin (Mmaso/Kukhutu) \| \| 39. Tetracycline (Mmaso) \| |  |
| 1. **Sorting Medicines activity two:** We would now like you to pick out the medicines that you have ever used before in your household. Please place them together in a pile.   ***Zochitika zachiwiri posanthula mankhwala:*** *Tsopano tifuna musankhe mankhwala amene munawagwirisapo ntchito pa banja lanu kuchokera pa mulu woyamba uja. Chonde ayikeni pa mulu umodzi*  ***Hint:*** *Remove unrecognised medicines (they’re now gone for good). Tip out recognised medicine and spread them out in front of the participant.*   \| 1. Amoxicillin (Mapilisi) \| \| --- \| \| 2. Amoxicillin (Amadzi) \| \| 3. Ampicillin (Mapilisi) \| \| 4. Azithromycin (Mapilisi) \| \| 5. Benzathene Penicillin (Obaya) \| \| 6. Benzylpenicillin (Obaya) \| \| 7. Ceftriaxone (Obaya) \| \| 8. Cefuroxime (Mapilisi) \| \| 9. Cefalexin (Mapilisi) \| \| 10. Cefixime (Mapilisi) \| \| 11. Chloramphenicol (Mapilisi) \| \| 12. Chloramphenicol (Obaya) \| \| 13. Ciprofloxacin (Mapilisi) \| \| 14. Clarithromycin (Mapilisi) \| \| 15. Clavulinic acid/ amoxicillin (Mapilisi) \| \| 16. Clindamycin (Mapilisi) \| \| 17. Clindamycin (Obaya) \| \| 18. Cloxacillin (Mapilisi) \| \| 19. Cloxacillin (Obaya) \| \| 20. Cotrimoxazole (Mapilisi) \| \| 21. Cotrimoxazole (Amadzi) \| \| 22. Doxycycline (Mapilisi) \| \| 23. Erythromycin (Mapilisi) \| \| 24. Erythromycin (Amadzi) \| \| 25. Flucloxacillin (Mapilisi) \| \| 26. Flucloxacillin/amoxicillin (Mapilisi) \| \| 27. Gentamycin (Obaya) \| \| 28. Levofloxacillin (Mapilisi) \| \| 29. Metronidazole (Mapilisi) \| \| 30. Metronidazole (Amadzi) \| \| 31. Metronidazole (Obaya) \| \| 32. Norfloxacin/ metronidazole (Mapilisi) \| \| 33. Ofloxacin/ Ornidazole (Mapilisi) \| \| 34. Phenoxymethylpenicillin (Mapilisi) \| \| 35. Tetracycline (Mapilisi) \| \| 36. Chloramphenicol (Mmaso/Kukhutu) \| \| 37. Ciprofloxacin (Mmaso) \| \| 38. Gentamicin (Mmaso/Kukhutu) \| \| 39. Tetracycline (Mmaso) \| |  |
| 1. **Sorting Medicines activity three:** We would now like you to pick out the medicines that you use frequently when someone in your household is sick. Please place them together in a pile.   ***Zochitika zachitatu posanthula mankhwala:*** *Tsopano tifuna kuti musankhe mankhwala amene mumawagwiritsa ntchito pafupipafupi kuchokera pa mulu wachiwiri pamene mmodzi wa pa banja panu wadwala. Chonde ayikeni pa mulu umodzi.*   \| 1. Amoxicillin (Mapilisi) \| \| --- \| \| 2. Amoxicillin (Amadzi) \| \| 3. Ampicillin (Mapilisi) \| \| 4. Azithromycin (Mapilisi) \| \| 5. Benzathene Penicillin (Obaya) \| \| 6. Benzylpenicillin (Obaya) \| \| 7. Ceftriaxone (Obaya) \| \| 8. Cefuroxime (Mapilisi) \| \| 9. Cefalexin (Mapilisi) \| \| 10. Cefixime (Mapilisi) \| \| 11. Chloramphenicol (Mapilisi) \| \| 12. Chloramphenicol (Obaya) \| \| 13. Ciprofloxacin (Mapilisi) \| \| 14. Clarithromycin (Mapilisi) \| \| 15. Clavulinic acid/ amoxicillin (Mapilisi) \| \| 16. Clindamycin (Mapilisi) \| \| 17. Clindamycin (Obaya) \| \| 18. Cloxacillin (Mapilisi) \| \| 19. Cloxacillin (Obaya) \| \| 20. Cotrimoxazole (Mapilisi) \| \| 21. Cotrimoxazole (Amadzi) \| \| 22. Doxycycline (Mapilisi) \| \| 23. Erythromycin (Mapilisi) \| \| 24. Erythromycin (Amadzi) \| \| 25. Flucloxacillin (Mapilisi) \| \| 26. Flucloxacillin/amoxicillin (Mapilisi) \| \| 27. Gentamycin (Obaya) \| \| 28. Levofloxacillin (Mapilisi) \| \| 29. Metronidazole (Mapilisi) \| \| 30. Metronidazole (Amadzi) \| \| 31. Metronidazole (Obaya) \| \| 32. Norfloxacin/ metronidazole (Mapilisi) \| \| 33. Ofloxacin/ Ornidazole (Mapilisi) \| \| 34. Phenoxymethylpenicillin (Mapilisi) \| \| 35. Tetracycline (Mapilisi) \| \| 36. Chloramphenicol (Mmaso/Kukhutu) \| \| 37. Ciprofloxacin (Mmaso) \| \| 38. Gentamicin (Mmaso/Kukhutu) \| \| 39. Tetracycline (Mmaso) \|  1. **Sorting Medicines activity four:** We would now like you to pick out any medicines that you have needed before in your household but you could not get. To say this another way, for each medicine here, has there ever been a time when you have needed the medicine but could not get it? Please place them together in a pile.   ***Zochitika zachinayi posanthula mankhwala:*** *Tsopano musankhe makhwala amene anakulemberani kapena analembera mmodzi wa a pa banja panu koma simunawapeze. Mukhozanso kunena kuti, pa mankhwala amene ali apawa, pali nthawi imene wa pa banja panu anamulembera koma mankhwalawo simunawapeze? Chonde ayikeni pa mulu umodzi.*  **Hint:** *Tip out the contents of all recognised medicines and spread them out in front of the participant.*   \| 1. Amoxicillin (Mapilisi) \| \| --- \| \| 2. Amoxicillin (Amadzi) \| \| 3. Ampicillin (Mapilisi) \| \| 4. Azithromycin (Mapilisi) \| \| 5. Benzathene Penicillin (Obaya) \| \| 6. Benzylpenicillin (Obaya) \| \| 7. Ceftriaxone (Obaya) \| \| 8. Cefuroxime (Mapilisi) \| \| 9. Cefalexin (Mapilisi) \| \| 10. Cefixime (Mapilisi) \| \| 11. Chloramphenicol (Mapilisi) \| \| 12. Chloramphenicol (Obaya) \| \| 13. Ciprofloxacin (Mapilisi) \| \| 14. Clarithromycin (Mapilisi) \| \| 15. Clavulinic acid/ amoxicillin (Mapilisi) \| \| 16. Clindamycin (Mapilisi) \| \| 17. Clindamycin (Obaya) \| \| 18. Cloxacillin (Mapilisi) \| \| 19. Cloxacillin (Obaya) \| \| 20. Cotrimoxazole (Mapilisi) \| \| 21. Cotrimoxazole (Amadzi) \| \| 22. Doxycycline (Mapilisi) \| \| 23. Erythromycin (Mapilisi) \| \| 24. Erythromycin (Amadzi) \| \| 25. Flucloxacillin (Mapilisi) \| \| 26. Flucloxacillin/amoxicillin (Mapilisi) \| \| 27. Gentamycin (Obaya) \| \| 28. Levofloxacillin (Mapilisi) \| \| 29. Metronidazole (Mapilisi) \| \| 30. Metronidazole (Amadzi) \| \| 31. Metronidazole (Obaya) \| \| 32. Norfloxacin/ metronidazole (Mapilisi) \| \| 33. Ofloxacin/ Ornidazole (Mapilisi) \| \| 34. Phenoxymethylpenicillin (Mapilisi) \| \| 35. Tetracycline (Mapilisi) \| \| 36. Chloramphenicol (Mmaso/Kukhutu) \| \| 37. Ciprofloxacin (Mmaso) \| \| 38. Gentamicin (Mmaso/Kukhutu) \| \| 39. Tetracycline (Mmaso) \| | (***This should be linked to the above question***) |
| 1. **Reason why they cannot get the antibiotic they need**   **Zifukwa zimene simungapezele mankhwala amene amumawafuna**  **It is not available in the government health facility**  **Sakupezeka mu zipatala zaboma**  **It is not available in the drug shop/pharmacy**  **Sakupezeka muzipatala za pulayiveti kapena malo wogulitsa mankhwala**  **It is too expensive**  **ndiwoodula**  **Other (Specify)**  **Zina (fotokozani)** |  |
| 1. **How often are the frequently used antibiotics used in the household? Kodi ndi mowirikiza bwanji momwe mumagwilitsa ntchito mankhwala omwe mumagwilitsa ntchito mowilikiza panyumba pano?**   Every day  Every week  Every two weeks  Every month  1-6 months  6-12 months  Other (Specify) |  |
| 1. **Sorting Medicines activity five:** We would now like you to pick out any medicines that you have used before in your household but they have not worked. To say this another way, for each medicine here, has there ever been a time where you have used this medicine in your household and it did not work? Please place them together in a pile.   *Tsopano tifuna kuti musankhe mankhwala amene inu kapena wa pa banja panu analembeledwa mankhwala amenewo koma mankhwalawo sanagwire ntchito mmene mumayembekezerera. Mukhoza kuwona kuti pa mankhwala ali apawa alipo amene nthawi ina anakulemberani koma mutamwa (inu/a pa banja panu) simunachire. Chonde ayikeni pa mulu umodzi.*   \| 1. Amoxicillin (Mapilisi) \| \| --- \| \| 2. Amoxicillin (Amadzi) \| \| 3. Ampicillin (Mapilisi) \| \| 4. Azithromycin (Mapilisi) \| \| 5. Benzathene Penicillin (Obaya) \| \| 6. Benzylpenicillin (Obaya) \| \| 7. Ceftriaxone (Obaya) \| \| 8. Cefuroxime (Mapilisi) \| \| 9. Cefalexin (Mapilisi) \| \| 10. Cefixime (Mapilisi) \| \| 11. Chloramphenicol (Mapilisi) \| \| 12. Chloramphenicol (Obaya) \| \| 13. Ciprofloxacin (Mapilisi) \| \| 14. Clarithromycin (Mapilisi) \| \| 15. Clavulinic acid/ amoxicillin (Mapilisi) \| \| 16. Clindamycin (Mapilisi) \| \| 17. Clindamycin (Obaya) \| \| 18. Cloxacillin (Mapilisi) \| \| 19. Cloxacillin (Obaya) \| \| 20. Cotrimoxazole (Mapilisi) \| \| 21. Cotrimoxazole (Amadzi) \| \| 22. Doxycycline (Mapilisi) \| \| 23. Erythromycin (Mapilisi) \| \| 24. Erythromycin (Amadzi) \| \| 25. Flucloxacillin (Mapilisi) \| \| 26. Flucloxacillin/amoxicillin (Mapilisi) \| \| 27. Gentamycin (Obaya) \| \| 28. Levofloxacillin (Mapilisi) \| \| 29. Metronidazole (Mapilisi) \| \| 30. Metronidazole (Amadzi) \| \| 31. Metronidazole (Obaya) \| \| 32. Norfloxacin/ metronidazole (Mapilisi) \| \| 33. Ofloxacin/ Ornidazole (Mapilisi) \| \| 34. Phenoxymethylpenicillin (Mapilisi) \| \| 35. Tetracycline (Mapilisi) \| \| 36. Chloramphenicol (Mmaso/Kukhutu) \| \| 37. Ciprofloxacin (Mmaso) \| \| 38. Gentamicin (Mmaso/Kukhutu) \| \| 39. Tetracycline (Mmaso) \| |  |
| 1. **Please tell me about your most recent experience using one of the antibiotics you frequently use to manage illnesses in your household?**   **Chonde tandiwuzeni zokhudzana ndi matenda omwe mwakumana nawo posachedwapa pakugwilitsa ntchito mankhwala omwe mumagwilitsa ntchito kawirkawiri pakuchiza matenda munyumba mwanu**  •         What happened that led you to use this medicine?  Kodi chinapangitsa kuti mugwilitse ntchito mankwalawa ndi chiyani?  •         How did you get to know about this medicine?  Kodi munawadziwa bwanji mankhwalawa amenewa?  •         Where did you get it from?  Kodi munawatenga kuti?  •         What did it cost you – in terms of money, time, etc?  Kodi munawapeza pamtengo wochuluka bwanji? ([ndipo zinakutengelani nthawi yochuluka bwanji?  •         What is it like to take the medicine?  Kodi zimakhala bwanji kumwa mankhwala?  •         How often is this medicine needed in this household?  Kodi ndi mowilikiza bwanji pamene mankhwala amenewa amafunika pa nyumba pano?  •         What would you do next time?[  Kodi mungadzapange bwanji nthaw yina?[follow up]  •         What happens when you can’t get an antibiotic you need?  Kodi mumachita chiyani pamene simunapeze mankhwala amene mumawafuna? | (**Provision for recording**) |
| **FARMING AND COMPANION ANIMAL CHARACTERISTICS we are now moving onto another topic of the interview. We would like to discuss any farming activities you undertake in the household**  **Tsopano tikupita gawo lina lakucheza kwathu. Tikufuna kuti tikambilane za ulimi umene mumachita pakhopmo pano** | |
| 1. **Does this household undertake farming activity for their own subsistence or income? (Farming includes livestock, poultry and agriculture) Kodi pakhomo pano mumapanga ulimi wa chakudya kapena zogulitsa?[ tikutathawuza ulimi waku munda kapena wa ziweto]**   Yes  No **(*If no, end interview here*)** |  |
| 1. **What types of farming activity do you undertake? (Tick all that apply) Kodi ndi mtundu wanji wa ulimi umene mumapanga?**   Crop farming  Livestock farming  Poultry  Other |  |
| 1. **What is the scale of farming activity? (Tick all that apply)**   **Kodi ulimi umene mumapangawo ndi wawukulu bwanji?**  Subsistence wachakudya  Commercialwokugulitsa  Other **zina** |  |
| 1. **What crops are grown? (Tick all that apply)**   **Kodi mumalima mbewu zanji?**  None  Maize  Sugar cane  Beans  Rice  Sweet potatoes  Cassava  Millet  Groundnuts  Other (Specify) |  |
| 1. **What livestock are kept? (Tick all that apply)** 2. **Kodi mumasunga ziweto zanji?**   None  Pigs  Cows  Goats  Sheep  Donkeys  Other (Specify) |  |
| 1. **What poultry are kept? (Tick all that apply)** 2. **Kodi mumasunga ziweto zanji ( zagulu la mbalame)**   None  Chicken  Turkey  Ducks  Pigeons  Guineafowl  Other (Specify) |  |
| 1. **Do you have any domestic animals such as dogs and cats?**   **Kodi muli ndi zoweta zinazili zones zapakhomo ngati galu ndi amphaka?**  Yes ____ dogs / no dogs  Yes ____ cats / no cats  Yes ___ other please specify |  |
| 1. **Duration the household has undertaken farming activity**   **Kodi mwapanga ulimi kwa nthawi yayitali bwanji?**  Months________ (if less than a year)  Years _________( if more than a year) |  |
| 1. **Who in the household is primarily responsible for farming activities? (Tick all that apply) Kodi pakhomo pano ndindani amene amayendetsa kapena kuyang’anila nkhani ulimi?**   Head of household  Primary caregiver  Child  Relative  Friend  Other (Specify) |  |
| **ILLNESS AND MEDICINES USED IN FARMING AND DOMESTIC ANIMALS** | |
| 1. **Commonly experienced illnesses in livestock farming** (***Tick all that apply)***   **kodi matenda anji amene amapezeka kwambili mu ziweto zag ulu la mbalame**   - Not applicable - Worms - Eye problems - African Swine fever - Swine Eryspelis - Caseous Lymphaditis - Black quarter - Foot and mouth disease - Mastitis - Sudden death - Skin disease/wounds - Diarrhoea - Typhoid - External parasites - Cough/Flu/Pneumonia - Other (Specify) |  |
| 1. **Commonly experienced illnesses in domestic animals** (***Tick all that apply)***   **Kod ndi matenda ati amene amapezeka kwambili mu ulimi wa ziweto zopezeka pa khom**   - Not applicable - Worms - Foot and mouth disease - Mastitis - Sudden death - Skin disease/wounds - Diarrhoea - Typhoid - External parasites - Cough/Flu - Other (Specify) |  |
| 1. **Commonly experienced illnesses in poultry**   (***Tick all that apply)***  ***Matenda amene amapezeka kwambili mu ziweto za gulu la mbalame***   - Not applicable - Diarrhoea - Worms - Typhoid - New castle disease - Coccidiosis - Chicken pox - Flu/cough - Sudden death - Skin disease/wounds - General weakness - External parasites - Other (Specify) |  |
| 1. **Commonly experienced diseases in agriculture** (***Tick all that apply) kodi ndi Matenda amene amapezeka kwambili mu ulimi***  - Not applicable - Maize weevil - Maize worms - Cassava mosaic - Cassava worms - Sweet potato worms - Rice worms - Banana weevil - Other (Specify) |  |
| 1. **When your animals are sick, what do you do?** (***Tick all that apply) Kodi ziweto zanu zikadwala mumapanga nazo bwanji?***  - Consult someone with experience   Timafunsa munthu amene ali ndi upangili   - Consult a qualified professional (Veterinarian or Agricultural officer) timafunsa mulangizi wa vetenale - Visit the Vet Drug shop vendor/Agro vet shop timapita ku malo wogulitsa mankhala azinyama - Other (specify) zina fotokozani |  |
| 1. **Where do you source your medicines for your animals?** (***Tick all that apply) Kodi mumapeza kuti mankwala azinyama zanu***  - Veterinary Drug shop - Veterinary Pharmacy - Agro veterinary Shop - Human medicines Pharmacy - Other farmers - Veterinary officer - Agricultural officer - Imported - Research clinic/NGO - Other (specify) |  |
| 1. **Can you show me any medicines you keep yourself for livestock, poultry or domestic animals?** (***Tick all that apply)***   **Kodi mungandiwonetseko mankhwala amene mumasunga a ziweto kapena ziweto zapakhomo**   - Medicines for Nagana - Medicines for general weakness - Medicines for typhoid - Medicines for Worms - Medicines for Foot and mouth disease - Medicines for Mastitis - Medicines for Skin disease/wounds - Medicines for Diarrhoea/bloody stool - Medicines for External parasites - Medicines for Cough/flu - Medicines for New castle disease - Medicines for Coccidiosis - Medicines for chicken pox - Medicines for constipation - Medicines for pain - Medicines for fever - Supplements (e.g vitamins) - Vaccines - Other (Specify) | ***(Provision for taking photo)*** |
| 1. **Can you show me any medicines you keep yourself for crops?** (***Tick all that apply) kodi mungandiwonetseko mankhwala ena aliwonse amene mumasunga ambewu?***  - Medicines for Maize weevil - Medicines for Maize worms - Medicines for Cassava mosaic - Medicines for cassava worms - Medicines for sweet potato worms - Medicines for Rice worms - Medicines for Banana weevil - Medicines for Other (Specify) | ***(Provision for taking photo)*** |
| 1. **Pile sorting activity 2. We have brought some medicines that are commonly used in livestock. We would like you to go through and show us the antibiotics you recognise** (***Tick all that apply) Takubweretserani mankhwala amene amagwiliotsidwa ntchito kawiri kawiri kuziweto tinakakonda kuti muwawone ndikutiwomnetsa mankhwala amene mukuwadziwa?***   **None (Skip to Qn 48)**  1. Oxytetracycline  2.Chlortetracycline hydrochloride  3. Doxycycline & Colistin sulphate  4. Procaine Penicillin & dihydrostreptomycin sulphate  5. Trimethoprim & Sulphamethoxazole BP  6. Amprolium & sulphaquinoxaline &vitamin k3  7. Amprolium hydrochloride  8. Sulfadimidine sodium  9. Norfloxacin 20%  10. Gentamicin Sulphate  11. Sulfadiazine & trimethoprim  12. Tylosin tartrate  13. Flumequine  14. enrofloxacin |  |
| 1. **Pile sorting activity 2. We would like you to return to this pile and pick out the antibiotics frequently used in livestock, poultry or domestic animals (*Tick all that apply)***   **Tikufuna kuti mubwelere pa mulu umenewu ndi kusankhapo mankhwala amene mumagwilitsa ntchito kawirikawiri ku ziweto ngati za mtundu wa mbalame ndi zinyama**  1. Oxytetracycline  2.Chlortetracycline hydrochloride  3. Doxycycline & Colistin sulphate  4. Procaine Penicillin & dihydrostreptomycin sulphate  5. Trimethoprim & Sulphamethoxazole BP  6. Amprolium & sulphaquinoxaline &vitamin k3  7. Amprolium hydrochloride  8. Sulfadimidine sodium  9. Norfloxacin 20%  10. Gentamicin Sulphate  11. Sulfadiazine & trimethoprim  12. Tylosin tartrate  13. Flumequine  14. enrofloxacin |  |
| 1. What conditions do you treat with each frequently used antibiotic in livestock, poultry or domestic animals?   Kodi ndi nthawi iti imene mumagwilitsa ntchito mankhwala wogwilitsidwa ntchito kawiri kawiriwa mu ziweto,ziweto zagulu la mbalame kapena zapakhomo (*Free list the condition that they treated against the medicine*)  1. Oxytetracycline  2.Chlortetracycline hydrochloride  3. Doxycycline & Colistin sulphate  4. Procaine Penicillin & dihydrostreptomycin sulphate  5. Trimethoprim & Sulphamethoxazole BP  6. Amprolium & sulphaquinoxaline &vitamin k3  7. Amprolium hydrochloride  8. Sulfadimidine sodium  9. Norfloxacin 20%  10. Gentamicin Sulphate  11. Sulfadiazine & trimethoprim  12. Tylosin tartrate  13. Flumequine  14. Enrofloxacin | ***(This should be linked to the above question)*** |
| 1. **Which, if any, of these frequently used antibiotics do you use in anticipation of sickness – without the animal becoming sick yet? Kodi ndi ati mwamankhwala omwe amagwilitsidwa ntchito kawirikawiriwa amagwilitsidwa ntchito pakukonzekera matenda, pa nthawi imene chiweto chisanadwale?**   1. Oxytetracycline  2.Chlortetracycline hydrochloride  3. Doxycycline & Colistin sulphate  4. Procaine Penicillin & dihydrostreptomycin sulphate  5. Trimethoprim & Sulphamethoxazole BP  6. Amprolium & sulphaquinoxaline &vitamin k3  7. Amprolium hydrochloride  8. Sulfadimidine sodium  9. Norfloxacin 20%  10. Gentamicin Sulphate  11. Sulfadiazine & trimethoprim  12. Tylosin tartrate  13. Flumequine  14. enrofloxacin |  |
| 1. **How often do you use the frequently used antibiotics you have identified here? Kodi ndi mowikiza bwanji pa nthawi imene mumagwilitsa ntchito mankhwala amene amagwila ntchito kawiri kawiriwa omwe mwa wa zindikira apawa?**   Every day  Every week  Every two weeks  Every month  1-6 months  6-12 months  Other (Specify) |  |
| 1. **Please tell me about your most recent experience using antibiotics to manage illnesses in farming in your household? Chonde tandiwuzeni zokhudzana ndi zimene mwakumana nazo pakugwilitsa ntchito mankhwala ndi cholinga choti muchize matenda mu ulimi pakhomo pano?**  - What happened that led you to use this medicine? - Kodi chinachitika ndi chiyani kuti mugwilitse ntchito mankhwala awa - How did you get to know about this medicine? - Kodi chinachitika ndi chiyani kuti mudziwe mankhwala awa - Where did you get it from? - Kodi munawatenga kuti? - What is it like to use the medicine in farming? - Kodi chiyembekezo chanu chimakhala chotani mukagwiritsa ntchito mankhwala mwatchulawa? - What did it cost you – in terms of money, time, etc? - Kodi zinakuwonongerani ndalama zingati, kapena nthawi yayitali bwanji? - What would you do next time? Kodi mudzapanga chiyani ulendo wina zitachitikanso? - What would it be like doing your farming activities without - antibiotics? Kodi zingakhale zotani kupanga ulimi opanda mankhwala ? | (***Provision for recording***) |
| 1. **Pile sorting activity 3. Of the recognised antibiotics, which have you needed for your animals but been unable to get? Pa mankhwala omwe mwawazindikirawa ndi ati omwe munawafuna kuti mugwilitse ntchitoku zinyama zanu koma simunathe kuwapeza**   1. Oxytetracycline  2.Chlortetracycline hydrochloride  3. Doxycycline & Colistin sulphate  4. Procaine Penicillin & dihydrostreptomycin sulphate  5. Trimethoprim & Sulphamethoxazole BP  6. Amprolium & sulphaquinoxaline &vitamin k3  7. Amprolium hydrochloride  8. Sulfadimidine sodium  9. Norfloxacin 20%  10. Gentamicin Sulphate  11. Sulfadiazine & trimethoprim  12. Tylosin tartrate  13. Flumequine  14. enrofloxacin |  |
| 1. **Can you tell me any reasons why you’ve been unable to get the antibiotic(s) you have needed? Mungathe kundifotokozela chifukwa chimene simunathele kupeza mankhwala amene munawafunawa**  - It is not available in the vet drug shop - It is too expensive - Other (Specify) |  |
