# Supplementary material for: Understanding antimicrobial use in subsistence farmers in Chikwawa District Malawi, implications for public awareness campaigns
Source: PLOS Glob Public Health. 2022 Jun 8;2(6):e0000314. doi: 10.1371/journal.pgph.0000314 (PMC10021458; doi:10.1371/journal.pgph.0000314)
Supplement: Attachment — Submitted filename: Responsetoreviewers.docx [file pgph.0000314.s003.docx]

**Responses to Reviewer 1**

1. This is an important paper on antimicrobial resistance drawing on empirical research to identify the life logics that shape the use and circulation of medicines in Malawi. The paper mobilises an anthropological approach, weaving together a combination of medicine interviews, ethnography and semi structured qualitative interviews to identify these dynamics. The paper makes the case that, without addressing systems issues, awareness campaigns may fail to exert their intended effect and work instead to reinforce inequalities and fail to protect the efficacy of medicines.

*We thank the reviewer for this comment and are very pleased to hear that they feel that this is an important paper.*

Specific questions and comments and listed below:

2. The authors described a public awareness image circulated in Malawi. How does circulation take place? Is it via WhatsApp, or other means? Greater detail would be useful here.

*The image was shared through WhatsApp, this has been made clearer in the paper see line 46-47 which reads:*

*“In Malawi, the image was being shared on WhatsApp, an instant messaging platform as part of the World Antibiotic Awareness Week, a global campaign coordinated by the World Health Organization.”*

3. Subsistence farmers are also described as being targets of awareness raising campaigns – how and where does this targeting take place?

*The awareness raising campaign was centred around World Antibiotic Awareness Week, through campaigns on the radio, TV as well as through mobile phone network providers.*

*We have modified the line to make it clearer that they are part of a broader campaign (see lines 152-153):*

*“We selected subsistence farmers in a low-income context because they are a group often economically marginalised yet still targeted with images and messages of awareness raising campaigns developed in the Global North.”*

4. Incorporating the image of the awareness campaign described in the introduction would help to bring the description to life.

*We had hoped to include this image in the manuscript, but unfortunately this is not in keeping with PLOS Global Public Health CC-BY 4.0 license.*

5. It felt at times that the authors could amplify further the significance of their findings by connecting their rich empirics to broader theory building efforts which demonstrate that interventions that are not in tune with context can not only fail to exert their intended effect but can also be accompanied by unintended consequences. Shove’s Beyond the ABC of Climate Change has sparked a lot of work in this regard and would be one of a number of relevant examples to connect to.

*Thank you for highlighting Shove’s important work. We have now cited her paper on ABC and climate change, and made more explicit the limits of a focus on individual behaviour change model in the introduction and discussion:*

*In the introduction (please see lines 91-95) which now reads:*

*“Parallels can be drawn between climate action and AMR in terms of the ways in which global policy has focused on individual behavioural change interventions, often overlooking the ways societal structures and economic models also require change (16,17). In these interventions, everyone is being urged to change their behaviour for the wider global good.”*

6. Line 50-51 – missing word ‘of’

*This has now been corrected.*

7. Where is Malawi situated in relation to poverty indicators?

*Malawi is one of the poorest countries in the world, with Chikwawa one of the poorest districts in the country.*

*We have now included this point (see lines 119-121) as follows:*

*“As one of the poorest countries in the world, and ranked 174 out of 189 countries in the Human Development Index,(29) urbanisation has been slower in Malawi than many other countries, and approximately eighty percent of population continue to reside in rural locations (30).”*

8. The materials and methods would benefit from addressing the following questions:

How long was data collection suspected during flooding?

*Data collection was suspended for 6 weeks, once fieldwork resumed, we worked in villages that were least affected. This detail has now been included (lines 201-202)*

*“When fieldwork restarted (6 weeks later), we carefully selected two sites for in-depth ethnographic work that had been least affected by the floods.”*

9. What did the day-to-day reach look like during the 6-month ethnography period – what did participant observation look like? How did the researchers keep notes during this time – were notes and observations written up daily?

*Thank you for this request for clarification, we have now provided further details in the paper (see lines 213-226)*

*“Building on relationships established during the structured medicine interviews, AN and ES spent time in the two villages, staying overnight and engaging with families primarily engaged in subsistence farming. Day-to-day they spent time in households and in communal spaces to develop a picture of livelihood and leisure activities. AN interacted more with men and ES with women. The broad focus of the study was to understand how people and households within the village responded to ill-health, and how medicines were accessed and used in this response. During initial community entry activities, this focus was emphasised during village meetings. AN and ES used the networks they established in the two villages to follow up with any household where a family member was sick, visiting the compound to understand the illness episode. They also spent time with the village health committee and at a health post based in one of the villages. Notes were hand-written at the end of each day in field diaries and then typed up at the end of each week.”*

10. The methodology lacks specificity re the coding process (e.g. did you identify first and second order codes and was this done by some or all members of the research team?). How did you enable your empirics to breathe and not to be over-determined by theory?

*We used a team approach to analysing the data. This began with weekly debriefing sessions with the research assistants to share early insights into the empirical data. As we moved through the analysis process, we also held two analysis workshops, ensuring that the whole data collection team were able to reflect on the coding frame and the themes as they were further developed. This approach allowed us to stay close to the data and not be over-determined by theory. We have now provided more in-depth discussion in the text (please see lines 235-252)*

*“We used a collaborative approach to analysing the ethnographic and interview data. During the six-month data collection period, we held weekly debriefing sessions with the team to reflect on data collection progress and processes. During these meetings we identified and addressed any ethical concerns encountered in the field and identified emerging themes and any new insights or avenues for enquiry to be followed up in the next week. We also built on work initially undertaken within the primary health care facilities. The development of the first coding frame took place iteratively, building on the reading of the data, and the debriefing sessions. All transcripts and fieldnotes were imported into NVivo 12 and the programme was used to aid data analysis by coding against the thematic framework. Data was coded in NVivo after the data collection was completed, with two further analysis workshops held virtually with the whole research team to allow for and reflection throughout the process.”*

11. The analysis section unpacks three broadly defined sets of relations that shape how and why people seek medicines outside of formal health care. It would be useful to clarify which (if any) of the themes were more prominent than others. Were there any relations or logics/minor themes that did not meet a coding threshold?

*The themes were interpreted as equally prominent and intersecting. Sub-themes sat within the themes that are presented in the paper and did not reveal additional or contradictory dimensions.*

12. Labelling of field notes and interview extracts – not always consistent - it wasn’t always clear if quoted participant material was from the same or a different participant. Quotes of empirics are made e.g. line 254-255 without reference to e.g. interviewee number.

*We had a different numbering system for the medicine interviews, interviews and fieldnotes. We have now ensured that details have been added to each participant including their occupation and gender.*

13. The analysis is well written and provides rich anthropological narrative. However, the start of the ‘doing the right thing section’ moves very quickly from a fieldnote extract to a statement about multiple ‘normative accounts.’ Engaging more empirical material prior to referring to ‘these normative accounts’ would help the transition from empirical richness to the analysis narrative.

*Thank you for raising this point, we have reviewed the manuscript and agree we make the statement about ‘normative accounts’ early in the theme. We do feel there is empirical material presented further in the theme, therefore we have reworked two paragraphs in this theme that read as follows*:

*P 13 lines 304-308:*

*“As can be seen in the fieldnote, when discussing experiences of navigating illness and seeking care, participants often conveyed narratives of the ‘right’ thing to do in terms of accessing and taking medicines. Participants talked of the importance of accessing ‘expert’ care through public health facilities, presenting symptoms and illness in the appropriate way, and accessing and taking medicines according to formal prescriptions only.”*

*P 15 lines 357-361:*

*“The narratives can be seen as normative accounts, through which participants conveyed the ‘right thing to do’ around care-seeking, and thus constructed themselves as good citizens, often in contrast with others. These normative accounts resonate with the focus of global health campaigns around AMR, such as the WHO co-ordinated World Antimicrobial Awareness Week (WAAW) (51).”*

14. The data is being made available via COMPASS but is not available yet and doesn’t accompany the submission.

*Data has been anonymised and will be held on LSHTM’s COMPASS. We have included all transcripts (in-depth interviews and structured medicine interviews) from the facility based and community-based components of the study. In line with our ethical approval, we have excluded raw fieldnotes due to the challenges in maintaining confidentiality.*

Thank you for the opportunity to review this engaging contribution.

**Response to reviewer 2**

1. The manuscript is very interesting and well written.
2. It will be good to see more information on the included households or health care professionals quotes (gender, age, education).

*We have now provided information on the gender of the participant. Given the geographical size of the area and that we name the villages we worked within, we decided not to include age and education as we were concerned about confidentiality.*

3. The author can link the issues presented within this manuscript to sustainable development goals and argue on how people of Chikwawa will not be left behind? because sustainable development is connected to global health, this will enrich the discussion on what would be the needed interventions and basic foundation for policy maker in order to intervene.

*Thank you for this comment*. *We agree that it is critical that AMR is more clearly linked to SDGs and that furthermore the interventions to address AMR can be better aligned in principle and practice to meet these wider goals. We have added this to the last paragraph of the discussion, page 26 lines 617-619, which now reads:*

*“.. and that communities have basic levels of security to cope with future illness and economic shocks. In this way, efforts to address AMR can align with wider efforts to meet sustainable development goals (SDGs).”*
