# Supplementary material for: Understanding antimicrobial use in subsistence farmers in Chikwawa District Malawi, implications for public awareness campaigns
Source: PLOS Glob Public Health. 2022 Jun 8;2(6):e0000314. doi: 10.1371/journal.pgph.0000314 (PMC10021458; doi:10.1371/journal.pgph.0000314)
Supplement: Attachment — Submitted filename: Response to reviewers.docx [file pgph.0000314.s004.docx]

Thank you for submitting this revised manuscript. I believe the reviewer noted limitations and recommendations for improvements have been addressed.

*Thank you, we are glad that you feel the reviewers comments have been adequately addressed.*

1. However, I have four methodological issues that need to be clarified by the authors. Please keep in mind I am well aware of the need to completed additional analysis with qualitative data. There are space limitations for manuscripts and multiple objectives in many larger studies, especially ethnographic and mixed methods. However, the manner in which this data is reported needs to be clearly, concisely, and transparently described. For the most part, the revised manuscript reads well but the four points below need to be further addressed, either through revisions or with clearly articulated arguments about why any of the revisions are not necessary. Again, the manuscript will quickly move forward with an acceptance once the next four points and a final recommendation are addressed. Thank you in advance for your patience and understanding.
   1. 1 - Please clarify the study design. There is not a study design stated.

*We have now clarified the study design at the start of the methods section.*

*Please see page 8 (lines 164-175)*

*“This study used an ethnographic methodology that was informed by the principles of critical medical anthropology, which emphasises the need to attend to the social, economic and political context of ill-health and healing (44). The study was conducted as part of the FIEBRE study, a multi-country and multidisciplinary investigation of febrile illness and antimicrobial use in five countries in Africa and Asia (45).* *The social science study in Malawi explored the emergent roles of antimicrobials in primary health care settings and in residential areas in Chikwawa. This paper reports the findings from the research with residents. The findings link to the research carried out in health facilities which are presented elsewhere (41). The study used a range of qualitative ethnographic methods (described below) to answer three questions (46) and sought to answer three research questions: (1) what are the most frequently used antibiotics in Chikwawa district? (2) How do subsistence farmers in rural Chikwawa seek medicines, care, and recover from illness? (3) How do antimicrobials and other medicines circulate in formal and informal settings in rural Chikwawa?”*

2. 2 - Please clarify the study as reporting either an analysis from a primary (original) data collection or secondary use of previously collected data. After reviewing the published protocol, a third less likely option is the original study was segregated into different segments for reporting (but I am not sure after my review of all the documents).

*Thank you for this request. The paper presents primary analysis of original data. The research, as described in the multi-country study protocol, aimed “to collect social science data on the roles of antimicrobials in fever case management for prescribers, local residents and a range of stakeholders.” This paper presents the findings from the research with local residents in Malawi. The findings of course interlink with the findings of the research with prescribers which is presented in a separate paper. We have clarified this in the paper on page 7-8 as follows,*

*“The social science study in Malawi explored the emergent roles of antimicrobials in primary health care settings and in residential areas in Chikwawa. This paper reports the findings from the research with residents. The findings link to the research carried out in health facilities which are presented elsewhere (41).”*

3. 3 - Please clarify the method of data analysis.
   *Our analytic approach was collaborative, inductive and drawing primarily on a thematic analysis approach. We have now updated the paper as follows (page 10-11 line numbers 252-269):
   Frequencies of antibiotic recognition and use in households were established based on the structured medicine interviews. Analysis of the data collected from the ethnographic fieldwork and in-depth interviews was conducted through a collaborative, inductive, iterative approach, drawing primarily on a thematic analysis approach (44). To ensure all team members contributed to the analysis, during the six-month data collection period, we held weekly debriefing sessions with the team to reflect on data collection progress and processes. During these meetings we identified and addressed any ethical concerns encountered in the field and identified emerging themes and any new insights or avenues for enquiry to be followed up in the next week. We also built on work initially undertaken within the primary health care facilities. The development of the first coding frame took place iteratively, building on the reviewing of the data, and the debriefing sessions. All transcripts and fieldnotes were imported into NVivo 12 and coded line-by-line to group ideas and develop themes to explain emergent phenomena. To complete the analysis two further analysis workshops held virtually with the whole research team to allow for and reflection throughout the process.*
4. 4 - Address the limitations inherent in this study design, especially if the data was a secondary analysis.

   *As noted in response 2, this paper presents primary analysis of original data. We have now provided further reflection on the limitations of this study design.

   Please see page 26 lines 634-640:

   Ideally the ethnographic fieldwork for the study would have run for longer than 6 months to allow for further deepening of relationships between the study team and residents in the two villages. However, due to the COVID-19 pandemic and the suspension of all field activities the study team stopped data collection. ES and AN have extensive experience of conducting health research in the area, which helped to contextualise the findings and supported the analysis helping to overcome some of this study limitation.*
5. ADDITIONAL INFORMATION
   1. - Yes, I am familair with critical medical anthropology, however this is not a study design. From what I understand, this was an ethnographic study, is this not correct? If correct, please state this to be the case at the beginning of the methods section with the appropriate methodological citations. If not correct, please justify the study design as written. Later in the methods section "anthropologically informed methods" is not appropriate to meaningfully address the question about study design.

   "This research was conducted as part of the FIEBRE study, a multi-country and multidisciplinary investigation of febrile illness and antimicrobial use in five countries in Africa and Asia (43). The social science study in Malawi, explored the roles of antimicrobials in both primary health care provision and within the community and utilised an ethnographic methodology that was informed by critical medical anthropology (44)."

*Thank you for this further detail, we have added methodological citations and provided more information outlining the study approach as noted above in response 1. Please see page 8 (lines 164-173)*

*“This study used an ethnographic methodology that was informed by the principles of critical medical anthropology, which emphasises the need to attend to the social, economic and political context of ill-health and healing (44). The study was conducted as part of the FIEBRE study, a multi-country and multidisciplinary investigation of febrile illness and antimicrobial use in five countries in Africa and Asia (45). The social science study in Malawi explored the emergent roles of antimicrobials in primary health care settings and in residential areas in Chikwawa. This paper reports the findings from the research with residents. The findings link to the research carried out in health facilities which are presented elsewhere (41). The study used a range of qualitative ethnographic methods (described below) to answer three questions (46) and sought to answer three research questions: (1) what are the most frequently used antibiotics in Chikwawa district? (2) How do subsistence farmers in rural Chikwawa seek medicines, care, and recover from illness? (3) How do antimicrobials and other medicines circulate in formal and informal settings in rural Chikwawa?”*

6. 2 - The study seems to be a secondary data analysis resulting from a larger ethnographic study. If this is the case, please revise the methods to clearly state this to be the fact with the citation for the larger study, and the protocol.
   "This paper reports primarily on the community-based research, but is informed by findings from research we conducted in the health care settings which are presented more fully elsewhere (45)."

*As noted above in response 2, the paper presents primary analysis of original data and but is interlinked with the data presented on care and prescribing practices published elsewhere. We think some of the confusion may have come from our presentation and description of findings of this study in the methods section. We have now provided a description of the study in the background section and provided greater clarity on the two studies.*

*For the text in the background please see page 7 lines 139-147*

*As described elsewhere, we have previously shown that in primary health care facilities in Chikwawa, care centred on the provision of an antimicrobial. Amid chronic lack of essential medicines and other resources, health workers relied on only a few medicines – with cotrimoxazole and lumefantrine/artemisinin (antimalarial) drugs provided by international donors being the most frequently prescribed and dispensed to patients. Clinical interactions were tightly scripted which provided patients with little time to ask questions or negotiate their treatment. Fever was conceptualised by participants as more than just a symptom of malaria, reflecting global health campaign messages that not all fever is malaria (41).*

*For text in the methods please see p 8 164-173:*

*“This study used an ethnographic methodology that was informed by the principles of critical medical anthropology, which emphasises the need to attend to the social, economic and political context of ill-health and healing (44). The study was conducted as part of the FIEBRE study, a multi-country and multidisciplinary investigation of febrile illness and antimicrobial use in five countries in Africa and Asia (45). The social science study in Malawi explored the emergent roles of antimicrobials in primary health care settings and in residential areas in Chikwawa. This paper reports the findings from the research with residents. The findings link to the research carried out in health facilities which are presented elsewhere (41). The study used a range of qualitative ethnographic methods (described below) to answer three questions (46) and sought to answer three research questions: (1) what are the most frequently used antibiotics in Chikwawa district? (2) How do subsistence farmers in rural Chikwawa seek medicines, care, and recover from illness? (3) How do antimicrobials and other medicines circulate in formal and informal settings in rural Chikwawa?”*

7. 3 - The data analysis is not clearly explained. What was the specific process for data analysis? The work done for the current study is not addressed. Was the analysis original from the secondary data? Was the data analyzed inductively, deductively, or perhaps both ways?

"Frequencies of recognition and use in households were established based on the structured medicine interviews. Analysis for the ethnographic fieldwork and in-depth interviews was conducted inductively through the course of the research. We used a collaborative approach to analysing the ethnographic and interview data. During the six-month data collection period, we held weekly debriefing sessions with the team to reflect on data collection progress and processes. During these meetings we identified and addressed any ethical concerns encountered in the field and identified emerging themes and any new insights or avenues for enquiry to be followed up in the next week. We also built on work initially undertaken within the primary health care facilities. The development of the first coding frame took place iteratively, building on the reading of the data, and the debriefing sessions. All transcripts and fieldnotes were imported into NVivo 12 and the programme was used to aid data analysis by coding against the thematic framework. Data was coded in NVivo after the data collection was completed, with two further analysis workshops held virtually with the whole research team to allow for and reflection throughout the process."

*As described in response 3. We have now provided further detail of the way in which the analysis was approached. Please see P10-11 lines 251-269:*

*Frequencies of antibiotic recognition and use in households were established based on the structured medicine interviews. Analysis of the data collected from the ethnographic fieldwork and in-depth interviews was conducted through an inductive, iterative analytic approach, drawing primarily on a thematic analysis approach (44). To ensure all team members contributed to the analysis, during the six-month data collection period, we held weekly debriefing sessions with the team to reflect on data collection progress and processes. During these meetings we identified and addressed any ethical concerns encountered in the field and identified emerging themes and any new insights or avenues for enquiry to be followed up in the next week. We also built on work initially undertaken within the primary health care facilities. The development of the first coding frame took place iteratively, building on the reading of the data, and the debriefing sessions. All transcripts and fieldnotes were imported into NVivo 12 and coded line-by-line to group ideas and develop themes to explain emergent phenomena. To complete the analysis two further analysis workshops held virtually with the whole research team to allow for and reflection throughout the process.*

8. 4 - The limitations section in the revised manuscript is not sufficiently descriptive about the limitations for this type of study. Please address the limitations in the study design, not merely the setting and context of the responses from participants.

   "In the Chikwawa district, the Malawi-Liverpool-Wellcome Trust Clinical Research Programme has a prominent and established presence in the region. Local communities readily recognise vehicles and researchers coming from the programme, and likely associate its work with a set of expectations around biomedical care and the duties of the participant. This may have shaped the presentation of public narratives of doing the right thing and should be interpreted in relation to the research context."

*As noted in response 4, we have now provided further details on study limitations. Please see page 26 lines 634-640:*

*Ideally the ethnographic fieldwork for the study would have run for longer than 6 months to allow for further deepening of relationships between the study team and residents in the two villages. However, due to the COVID-19 pandemic and the suspension of all field activities the study team stopped data collection. ES and AN have extensive experience of conducting health research in the area, which helped to contextualise the findings and supported the analysis helping to overcome some of this study limitation.*

9. RECOMMENDATION -----

   Finally, I would like the authors to consider highlighting the aim of the previously published study:

   "We highlight the contours of what we refer to as antibiotic vulnerabilities, exploring what constitutes care within primary health care facilities in Chikwawa District, in Malawi and how this relates to antimicrobial prescribing."

   THEN, link this to the extension of this point in the aim of this study:

   "The aim of our study was to provide an in-depth understanding of the ways medicines were accessed and used by households dependent on subsistence farming in the Chikwawa District of Malawi."

   This would clearly explain this work resulted from a larger project and help readers to understand what is added by the current study.

*Thank you for raising this point. As noted in our response to point 2 above we have now provided further details of our study and how it relates to the data collected on care and prescribing practices. The work we present in this paper is primary analysis of original data.*
